# Assessment of the Antivirulence Potential of *Tamarix aphylla* Ethanolic Extract against Multidrug-Resistant *Streptococcus mutans* Isolated from Iraqi Patients

**DOI:** 10.1155/2023/6662262

**Published:** 2023-08-04

**Authors:** Sahira A. Aljubouri, Khalid H. Alobaidi

**Affiliations:** Department of Plant Biotechnology, College of Biotechnology, Al-Nahrain University, Baghdad 10001, Iraq

## Abstract

Halophytes have long been used for medicinal purposes. However, their use was entirely empirical, with no knowledge of the bioactive compounds. The plant *Tamarix aphylla* L. has not drawn the deserving attention for its phytochemical and bioactive explorations, but available data expressed its needs to be attended for its potential. The *Streptococcus mutans SpaP* gene (cell-surface antigen) mediates the binding of these bacteria to tooth surfaces. The growing problem of antibiotic resistance triggered the research on alternative antimicrobial approaches. Our study aims to explore the activity of *T. aphylla* ethanolic extract against the virulence gene found in *Streptococcus mutans* pathogenic bacteria. Samples that were previously collected and identified in our previous work (in press) were obtained from different dental clinics and hospitals in Baghdad. Three nonbiofilm-forming bacterial isolates having multidrug resistance (MDR) for 10 antibiotics (doxycycline, ofloxacin, tetracycline, erythromycin, vancomycin, clindamycin, rifampicin, imipenem, amikacin, and cefepime) were selected to examine the potential of the *T. aphylla* ethanolic extract. The ethanolic extract showed high antimicrobial activity against MDR. Minimum inhibition concentration (MIC) for the extract was 17.5 mg/ml, while minimum bactericidal concentration (MBC) was 35 mg/ml. The phytochemical compounds present in the ethanolic extract were determined by using high-performance liquid chromatography (HPLC) which revealed that the leaves contain thirteen different alkaloids, twelve flavonoids, and four vitamins. The extract strongly inhibited a virulence property, the adherence of *S. mutans* which reduced during critical growth phases. The one-step RT-PCR method was used to study the *SpaP* gene expression of bacterial isolates which significantly reduced. In conclusion, extraction of *T. aphylla* leaves showed an antimicrobial effect against MDR *S. mutans*. The identified phytochemicals in the *T. aphylla* extract are reported to be biologically important and need further investigation to develop safe and cheap drugs.

## 1. Introduction

For thousands of years, many illnesses have been treated with natural product-based medicine [1, 2]. Herbal remedies have long been essential in the development of medications to treat a range of disorders. Currently, medicines are variations or replicas of components, mixtures, and dilutions found in nature [3]. Due to fewer or no adverse effects, people are becoming more interested in herbal medications [4]. Even if there is a little chance of side effects, drug interaction is still a possibility. Cost-effectiveness is another motivating factor for approving a wide range of herbal medicines. The use of herbal remedies has a long history, but alternative medicine and complementary therapies that include cutting-edge technology are filling a gap in long-standing traditional practices [5, 6]. Because nature is a large resource, we should keep looking into new plant-based therapies.

More than 60 species of halophyte plants belonging to the genus *Tamarix* are cultivated in almost every region of the world under the popular names “Tamarisk” and “salt cedar” in the Tamaricaceae family. These plants are distinguished by their needle-like leaves that are covered with salt that the salt glands secrete [7]. Tamarisk species are well known for their growth in hot and dry climates; however, they are also found in temperate climates. *Tamarix* species are cultivated in dry climates in order to fix sand dunes [8]. As invasive plants, they inhibit the establishment of other species in moist climates; therefore, their presence there is undesirable [9]. Numerous phytochemical studies on various *Tamarix* species have identified a number of phytochemicals, the most significant of which are polyphenolic substances such as phenolic acids, flavonoids, and tannins. In addition, locals use tamarisk for therapeutic purposes in several Asian and African nations where it grows natively, including Pakistan, India, Iran, and Algeria [10].

According to nonclinical research, *Tamarix aphylla* exhibits considerable antibacterial, antioxidant, and cytotoxic properties. More research on extraction, separation, and purification is therefore required in order to create new medications that could treat a variety of illnesses. The results of numerous investigations demonstrated that the *T. aphylla* leaf extract is harmless, since there have been no reported fatalities, and studies from Saudi Arabia and Pakistan revealed that *T. aphylla* toxicity is minimal. Findings from the study suggest that *T. aphylla*'s pharmacological characteristics justify its conventional uses. The efficacy and safety of *T. aphylla* in humans must therefore be confirmed by high-quality preclinical research and well-planned clinical investigations, both of which were not found to exist [11].

One of the most prevalent health issues in the world in 2015 is dental caries, a chronic infectious illness that affects mineralized tooth tissue and places a significant financial, social, and health-related burden on society [12]. Dental caries has been linked to *Streptococcus mutans* as the main bacterial cause. In addition to its potential for acidogenesis, its capacity to stick to teeth and create a biofilm is a factor in its cariogenicity [13, 14]. *SpaP*, also known as P1, antigen I/II, or Pac, is a conserved sucrose-independent adhesin that is a key component of *S. mutans*' adherence and colonization. By directly interacting with salivary agglutinin (SAG), a salivary component, it facilitates the adhesion of *S. mutans* to the saliva-coated tooth surface [15].

*Streptococcus mutans* was isolated and identified from several dental caries clinical samples in Iraq to investigate and evaluate the antibacterial activity of widely available and abundant plants of *Tamarix aphylla* against *Streptococcus mutans* and its virulence factor *SpaP*, to produce natural green biocides with similar or higher performance compared with existing antibiotics.

## 2. Materials and Methods

### 2.1. Sample Selection and Collection

Samples that were previously collected and identified in our previous work at Al-Nahrain University/College of Biotechnology/Microbiology Laboratory (in press) were obtained from different dental clinics and hospitals in Baghdad. These bacterial isolates were used for molecular PCR identification. After molecular PCR identification and antibiotic sensitivity test (AST) were performed, only three multidrug-resistant bacteria were chosen to test plant products against it. The samples were stored in glycerol; by diluting 100% glycerol in distilled water, a glycerol solution was created. Bacterial growth in brain heart infusion broth was observed after an overnight period. A 2 mL cryovial was filled with 500 *μ*l of 50% glycerol and 500 *μ*l of broth after the overnight culture was added. The glycerol stock tube was frozen at −80°C. A portion of the frozen microorganisms was recovered from a glycerol stock by scraping them off the top using a sterile loop. The bacteria were then kept at 37°C for overnight incubation in BHI [16].

### 2.2. Antibiotic Sensitivity Test

Synthetic antibiotics were tested against pathogenic microorganisms, namely, *Streptococcus mutans*, using the Mueller–Hinton Agar. The disk diffusion method is used for ten antibiotics (doxycycline 30 mcg, ofloxacin 5 mcg, tetracycline 30 mcg, erythromycin 15 mcg, vancomycin 30 mcg, clindamycin 2 mcg, rifampicin 5 mcg, imipenem 10 mcg, amikacin 30 mcg, and cefepime 10 mcg). The pathogenic bacteria were streaked on the Petri dish. The Petri dishes were incubated at 37°C. After 24 h of incubation, for each microbial culture, the inhibition zones were measured in millimeters (mm) of the diameter of the impregnated disc included in the diameter of the halo [17].

### 2.3. Molecular Experimental Studies

Various steps were performed to conduct the molecular detection of *S. mutans* bacteria as follows.

#### 2.3.1. DNA Extraction

The following procedures were used to extract genomic DNA from bacterial growth using the ABIOpure extraction protocol: 1 ml of the culture was spun for 2 minutes at 13,000 rpm to obtain pellet cells. The excess liquid was then discarded. The sample was added to a tube along with 20 *μ*l of Proteinase K solution (20 mg/ml) and 200 *μ*l of buffer BL. The tube was then forcefully agitated using a vortex and incubated at 56°C for 30 min. 200 *μ*l of 100% ethanol was added to the sample, and the sample was thoroughly mixed using a pulse vortex. After carefully transferring each mixture to the small column, it was centrifuged for one minute at 8,000 rpm, and the collecting tube was changed for a fresh one. 600 *μ*l of buffer BW was added to the small column, which was then centrifuged for one minute at 8,000 rpm with a fresh collecting tube. TW 700 *μ*l was applied from the buffer at 8,000 rpm for 1 minute for centrifuging. The minicolumn was reinserted into the collection tube after the pass-through was discarded. The minicolumn was centrifuged at full speed for one minute (13,000 rpm) to remove any remaining wash buffer, and it was then put into a new 1.5 ml tube. 60 *μ*l of buffer AE was added, let to sit at room temperature for 1 minute, and then centrifuged for 5 minutes at 5,000 rpm.

#### 2.3.2. Quantitation of DNA

The concentration of extracted DNA was measured using a Quantus fluorometer to determine the sample quality for subsequent use. 200 *μ*l of diluted QuantiFluor dye was combined with 1 *μ*l of DNA. DNA concentration readings were found following a 5-minute incubation period at room temperature. The Macrogen Company provided these primers in lyophilized form. As a stock solution, lyophilized primers were dissolved in nuclease-free water at a final concentration of 100 pmol/*μ*l. 90 *μ*l of nuclease-free water was combined with 10 *μ*l of the primer stock solution (kept at a −20 C freezer) to create a useable primer solution containing 10 pmol/*μ*l of these primers.

#### 2.3.3. Reaction Setup and Thermal Cycling Protocol

*(1) PCR Amplification*. Isolated genomic DNA obtained from bacterial isolates was used as a template for PCR. *SpaP* and *vicR* genes were amplified. The PCR reaction was performed by adding 10 *μ*l of GoTaq® G2 Master Mix, 4 *μ*l of template DNA (genomic DNA), 1 *μ*l of each of the upstream and downstream primers, and 4 *μ*l nuclease-free water to complete the volume to 20 *μ*l. The reaction program consisted of the following steps: a first phase of 5 minutes of denaturation at 95 degrees Celsius and then 30 cycles of denaturation at 95 degrees Celsius for 30 seconds, annealing at 60 degrees Celsius for 30 seconds, and extension at 72 degrees Celsius for 30 seconds. There was also a final extension phase lasting 7 minutes at 72°C [18].

*(2) Agarose Gel Electrophoresis*. Agarose gel electrophoresis was used to verify the existence of amplification following PCR amplification. The criteria based on the isolated DNA were totally reliable for PCR.

- Solutions
- The solutions used were DNA ladder marker, 1X TAE buffer, and ethidium bromide (10 mg/ml).
- Preparation of Agarose
- 100 ml of 1X TAE in a flask was taken. The buffer received 1.5 g (for a 1.5% agarose concentration). The solution was microwaved to boiling when all of the gel particles were dissolved. To agarose, 1 *μ*l of ethidium bromide (10 mg/ml) was added. To combine agarose and prevent bubbles, it was stirred. The solution was allowed to cool at 50–60°C.
- Casting of the Horizontal Agarose Gel
- After sealing the edges with cellophane tape on both sides, the agarose solution was poured into the gel tray, where it was allowed to set for 30 minutes at room temperature. The gel was carefully placed in the gel tray after the comb had been removed. 1X TAE-electrophoresis buffer was poured into the tray until it was 3–5 mm above the gel's surface.
- DNA Loading
- The PCR products were directly loaded. 5 *μ*l of the PCR product was added straight to the well. 100v/mAmp of electricity was turned on for 60 minutes. DNA travels from the cathode to the positive anode poles. Using the gel imaging equipment, the ethidium bromide-stained bands in the gel were seen.

### 2.4. Plant Collection and Identification

Leaves of *Tamarix aphylla* were collected in September–November 2022 from the areas around the vicinity of The University of Wasit which is located in Al-Kut city, Wasit Governorate, Iraq, and were identified by expert taxonomist Dr. Sukaina A. Ehlaiwe, Field Crops Department College of Science, Baghdad University Herbarium, Iraq.

### 2.5. Plant Extract Preparation

The plant samples were thoroughly washed twice with running tap water and once with sterile water and then left at room temperature for three to five days in the dark. After being dried and ground into a fine homogenized powder in a grinder, the powder was then passed through a 0.5 mm·mesh screen before being stored in sterile polythene bags in the laboratory.

#### 2.5.1. Ethanolic Extraction

The plant leaves were dried for three weeks at room temperature. The plant's dried leaves were powdered in an electric grinder. 100 g of the powdered plant material was steeped in conical flasks containing 500 ml of 99.9% pure ethanol for 24 hours, and then, the extract was filtered to eliminate any insoluble materials using gauze and Whatman No. 1 filter paper. After filtration, the extract was put in a rotary evaporator instrument for 3 h at 50°C and 90 rpm speed, and after that, it was put in a Petri dish glass for drying using an oven at 40–50°C until it was entirely dried [19].

#### 2.5.2. Antibacterial Activity Test of Plant Extracts

The antibacterial activity of the crude extracts was examined using the well diffusion method as a preliminary screening procedure against the isolated *S. mutans*. The bacteria were added to an enrichment medium and incubated at 37°C until they reached the estimated density of a bacterial suspension that had been diluted and well combined to match the turbidity of the 0.5 McFarland standard (1.5 × 10^8^ CFU/ml), and they were streaked on MHB [20].

#### 2.5.3. Well Diffusion Test

A sterile cotton swab was dipped in the bacterial suspension and used to inoculate the Mueller–Hinton agar (pH 7.2) and 4 mm deep plates with *S. mutans*. To ensure a uniform distribution of inoculum, the prepared Mueller–Hinton agar plates were inoculated by dabbing the swab over the entire surface. A sterile cork borer was used to aseptically create a circular well with 6 mm diameter. The wells were then injected with 100 *μ*l of crude *T. aphylla* extracts at concentrations of 35 mg/ml, 70 mg/mL, and 140 mg/mL. As a negative control, 100% dimethyl sulfoxide (DMSO) was added to the well and incubated for 24–72 hours at 37°C. There were three copies of each experiment.

#### 2.5.4. Determination of Minimum Inhibitory Concentration (MIC) and Minimum Bactericidal Concentration (MBC)

The resazurin-based turbidimetric assay was used to determine MIC, while the broth dilution susceptible assay was used to determine MBC [21].

- Determination of Minimum Inhibitory Concentration (MIC) Using the Resazurin-Based Turbidimetric *Assay*
- Bacterial stock cultures (S. mutans) were subcultured onto Mitis Salivarius agar media and incubated overnight at 37°C. Next day, the McFarland standard (0.5McF) was required for the test using DensiCHEK Plus Meter.
- To make the resazurin solution, 337.5 mg of resazurin powder was dissolved in 50 ml distilled water. To ensure homogeneity, the solution was mixed for 1 hour using a sterile roll mixer. The preparation processes were carried out in complete darkness, and the resazurin solution was then stored in an amber glass container to avoid light exposure.
- The turbidimetric test based on resazurin was used to show the inhibitory effects of the Tamarix aphylla alcohol extract against S. mutans. One 96-well flat-bottom microtiter plate for the alcohol extract was prepared. The test consisted of one vertical row of broth used to check for sterility and three vertical rows for each microorganism. Each vertical row well received 100 *μ*l of Mueller–Hinton broth (MHB). The first well contained 100 *μ*l of plant extract products at a concentration of 280 mg/ml. The first well's mixture was completely mixed. Following that, using a separate sterile pipette, 100 *μ*l of the first well's mix was transferred to the second well (140 mg/ml) and mixed well. Following that, 100 *μ*l of the mixture was transferred from the second to the third well (70 mg/ml) and mixed again. This procedure was repeated until the tenth well was reached (0.2 mg/ml). Finally, 100 *μ*l was taken and discarded from the tenth well. Only 100 ml of Mueller–Hinton broth (MHB) is included in the last well. The final concentration of plant extract products was halved in each well. Then, 10 *μ*l of diluted bacterial suspension (1.5 × 108 cell/ml) was added to each well and mixed well. Following overnight incubation at 37°C, 5 *μ*l of resazurin (6.75 mg/ml) was added to each well and incubated for an additional 4 hours at 37°C. Changes in color were noticed and recorded. The concentration at which the color change occurred was taken as the minimum inhibitory concentration (MIC).
- Determination of Minimum Bactericidal Concentration (MBC)
- To ascertain the minimum bactericidal concentrations that were effective, working solutions of crude extracts (250 mg/mL) were serially diluted in the vials to make a group of dual concentrations of 8.75, 17.5, 35, 70, and 140 mg/mL in 4.5 ml of sterile tubes of the Muller–Hinton broth medium. S. mutans was grown in enrichment media for 2 days at 37°C. For two days, the tubes were incubated at 37°C. Then, from each tube, 100 *μ*l was cultured in the Muller–Hinton agar media for 2 days. When the lowest amount of crude extraction was added, which did not cause growth at the agar media, the minimum bactericidal concentration (MBC) effect was identified.

#### 2.5.5. Determination of Crude Extract Active Compounds Using High-Performance Liquid Chromatography (HPLC)

For HPLC analysis, 5 g of the alcoholic extract was dissolved in 10 ml of 70% absolute ethanol followed by filtration using a 0.22 mm millipore filter. The sample was analyzed by high-performance liquid chromatography (HPLC) to identify the active component of *Tamarix aphylla* [22]. The HPLC model used was Sykam S 2100.

 
*A* = Total alkaloid
  The mobile phase:
  ACN:glacial acetic:triethyl amine/97.9 : 2:0,1
  Gradient program:
  Flow rate = 1 ml/min
  Detector = 284 nm
  Temperature = room temperature
  Volume injected = 20 *μ*l
  Column = c18
  Total flavonoid:
  The mobile phase:
  0.1% H_3_PO_4_ in D. W.
  Gradient program:
  Flow rate = 0.8 ml/min
  Detector = 210 nm
  Temperature = room temperature
  Volume injected = 20 *μ*l
  Column = c18
  Total vitamins:
  The mobile phase:
  MEOH:H_2_O—35 : 65
  Gradient program:
  Flow rate = 1 ml/min
  Detector = 254 nm
  Temperature = room temperature
  Volume injected = 20 *μ*l
  Column = c18

The concentration for each *Tamarix aphylla* compound was calculated as follows:

(1) $$\begin{array}{c} The\;concentration\;of\;the\;sample=\left(\frac{area\;of\;the\;sample}{area\;of\;the\;standard}\right)\times concentration\;of\;the\;standard\times \;\frac{ml}{g}\text{.} \end{array}$$

### 2.6. Effect of Sub-MIC Concentration for the Ethanol Extract on the Bacterial Adhesion Assay

The technique assessed glass surface adhesion. In a nutshell, the bacteria were cultured in a glass tube containing 10 ml of BHI with 5% (w/v) sucrose and sub-MIC concentrations of the extracts (8.7 mg/ml) for 24 hours at 37°C and a 30° angle. BHI with (sucrose-dependent) and equal concentrations of DMSO and ethanol served as the solvent controls. Planktonic cells were delicately removed from the glass tubes after incubation. Then, once 0.5 M of NaOH was added, vortexing was used to remove the adherent cells. The cells were cleaned and placed in saline suspension. At 600 nm, adhesion was measured spectrophotometrically [23]:

(2) $$\begin{array}{c} Percentage\;adherence=\left(\frac{O.D.\;of\;adhered\;cells}{O.D.\;of\;total\;cells}\right)\times 100\text{.} \end{array}$$

### 2.7. Effect of Sub-MIC Concentration on *SpaP* Gene Expression

For each bacterial isolate, two tubes were used one for treatment with the plant extract (8.7 mg/ml) and the other used as a control without the plant extract; after 24 h incubation at 37°C, RNA was extracted.

#### 2.7.1. RNA Purification

The following procedures were used to extract RNA from the sample in accordance with the TRIzol^TM^ reagent protocol.

*(1) Sample Lysis*. In the suspension, bacterial cells were cultivated. For pellet cells, 1 ml of the cell culture was centrifuged for 2 min at 13,000 rpm to precipitate the cells, after which the supernatant was discarded and 0.75 mL of TRIzol^TM^ reagent was added to the pellet. Through repeated pipetting up and down motions, the lysate was homogenized.

*(2) For Three Phases Separations*. Each tube's lysate was mixed with 0.2 mL of chloroform before the tube cap was put on. All mixtures were incubated for 2-3 minutes and then separated into a colorless upper aqueous phase, an interphase, and a lower organic phase by centrifugation for 10 minutes at 12,000 rpm. The RNA-containing aqueous phase was moved to a fresh tube.

*(3) For RNA Precipitation*. The aqueous phase was supplemented with isopropanol (0.5 mL), which was then incubated for 10 minutes before being centrifuged at 12,000 rpm for 10 minutes. A pellet of white, gel-like precipitated total RNA formed at the tube's bottom. The supernatant was then thrown away.

*(4) For RNA Washing*. Each tube received 0.5 mL of 70% ethanol, which was added, quickly vortexed, and then centrifuged for five minutes at 10,000 rpm. After aspirating ethanol, the pellet was dried in air.

*(5) For RNA Solubility*. The pellet was rehydrated in 20–50 *μ*l of nuclease-free water before being incubated for 10–15 minutes in a water bath or heat block with a temperature setting of 55–60°C.

#### 2.7.2. Determine RNA Yield by the Fluorescence Method

In order to assess the quality of samples for use in later processes, the concentration of extracted RNA was detected using a Quantus fluorometer. 200 *μ*l of diluted QuantiFluor dye was combined with 1 *μ*l of RNA. RNA concentration values were found following a 5-minute incubation period at room temperature and in the dark.

#### 2.7.3. Primer Preparation

The Macrogen Company supplied these primers as a lyophilized stock solution, which was subsequently diluted in nuclease-free water to a final concentration of 100 pmol/*μ*l. The working solution for these primers was created by diluting 10 *μ*l of the primer stock solution, which was maintained at −20°C, in 90 *μ*l of nuclease-free water to yield a 10 pmol/*μ*l working solution (Table 1).

#### 2.7.4. Real-Time Reverse Transcribing PCR (RT-PCR)

Isolated RNA obtained from the bacterial isolates was used as a template for RT-PCR (SpaP and 16S RNA primer). The RT-PCR reaction was performed by adding 5 *μ*l of GoTaq® G2 Master Mix, 1 *μ*l of RNA template, 0.5 *μ*l of 10 pmol forward and reverse primers, 0.25 *μ*l GoScript™ Reverse Transcriptase, and 2.5 *μ*l nuclease-free water to complete the volume to 10 *μ*l in a 0.2 ml tube. The amplification program was as follows: cDNA synthesis step at 37°C for 15 min, this process starts with a five-minute initial denaturation stage at 95°C, followed by 40 cycles of denaturation at 95°C for 20 seconds, annealing at 60°C for 20 seconds, and extension at 72°C for 20 seconds.

#### 2.7.5. Relative Quantification Equation

(3) $$\begin{array}{c} Folding=2-\Delta \Delta CT\text{,}\\ \Delta CT=CT\;gene-CT\;housekeeping\;gene\text{,}\\ \Delta \Delta CT=\Delta CT\;treated\;or\;control-\Delta CT\;control\text{.} \end{array}$$

### 2.8. Statistical Analyses

Statistical analyses and reporting of the obtained data were carried out by using the computerized database structure. One-way analysis of variance (ANOVA) was used for more than two groups, and *P* < 0.05 was the accepted statistical significance.

## 3. Results and Discussion

### 3.1. Plant Collection and Identification

Leaves of *Tamarix aphylla* were collected in September–November 2022 from the areas around the vicinity of The University of Wasit which is located in Al-Kut city, Wasit Governorate, Iraq, and were identified by expert taxonomist Dr. Sukaina A. Ehlaiwe, Field Crops Department College of Science, Baghdad University Herbarium, Iraq.

### 3.2. Plant Extract Preparation

Extraction is the first step in the process of obtaining active compounds from the selected plants, which enable the isolation and derivation of bioactive compounds from secondary metabolites of plants. Extraction worked by using yields of a quantity of 9.79 g from the coarse powdered areal parts 100 g, representing 90% of the *T. aphylla* original sample.

#### 3.2.1. Ethanolic Extract Antibacterial Activity

**Figure 1:**
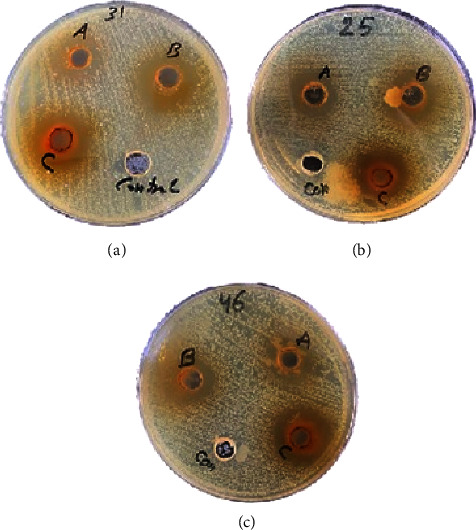
The inhibition zone of different concentrations of the crude *T. aphylla* ethanolic extract against 3 *S. mutans* isolates: (a) 35 mg/mL, (b) 70 mg/mL, and (c) 140 mg/mL and (control) 5% dimethyl sulfoxide.

The well diffusion method was used to determine the antibacterial activity. Three different concentrations (35 mg/mL, 70 mg/mL, and 140 mg/mL) of each plant crude extract were prepared by adding 5% dimethyl sulfoxide (DMSO) as polar solvent (Figure 1).

The ethanolic crude extract showed remarkable results with a clear inhibition zone around each well, and DMSO as a negative control showed no inhibition zone. The results showed an increasing size of the inhibition zone by increasing the concentration.

**Figure 2:**
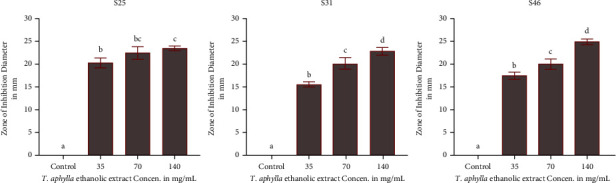
The significance difference in the zone of inhibition between the three *S. mutans* isolates S25, S31, and S46.

From the results shown in Figure 2, we can see that there are three individual treatments of the plant; the results demonstrated that all concentrations led to a significant increase (*P* ≤ 0.05) in the inhibition zone of *S. mutans* compared with a negative control (DMSO). There was a significant difference in the mean inhibition zone caused by the concentrations of the *T. aphylla* ethanolic extract. The interaction of a concentration of 140 mg/ml of *T. aphylla* resulted in up to a 24 mm inhibition zone; this value was significantly different from the lowest concentration (35 mg/mL), which resulted in an 18 mm inhibition zone. These findings are consistent with those of [24] who reported that crude extracts of *T. aphylla* demonstrated antibacterial activity and inhibition effects against both Gram-positive and Gram-negative bacteria and have promising antibacterial activity and can be a new natural source for bioactive compounds.

#### 3.2.2. Determination of Minimum Inhibitory Concentration (MIC) and Minimum Bactericidal Concentration (MBC)

The resazurin-based turbidimetric assay was used to determine MIC, while the broth dilution susceptible assay was used to determine MBC.

The results of the turbidimetric test based on resazurin were used to show the inhibitory effects of the *Tamarix aphylla* ethanolic extract against *S. mutans*. One 96-well flat-bottom microtiter plate for the ethanolic extract was prepared. The MIC for the *T. aphylla* ethanolic extract was determined and confirmed by subculturing the broth of the determined concentration on agar media. Minimum inhibition concentration (MIC) for the ethanolic extract was 17.5 mg/ml.

The minimum bactericidal concentration (MBC) of the crude extract was determined using the broth dilution method. The minimum bactericidal concentration (MBC) for *T. aphylla* was determined and confirmed by subculturing the last clear broth of MBC on agar media. The minimum bactericidal concentration (MBC) was 35 mg/ml.

#### 3.2.3. Determination of Crude Extract Active Compounds Using High-Performance Liquid Chromatography (HPLC)

In HPLC, qualitative identifications have been made by comparison of the retention times obtained at identical chromatographic conditions of analyzed samples with the authenticated reference standards. The HPLC analysis of the *T. aphylla* ethanolic extract determined that the leaves contained twelve flavonoids (higher concentration was 4-hydroxybenzoic 40.07 ppm) (Table 2), thirteen alkaloids (higher concentration was amfepramone 67.73 ppm) (Table 3), and four vitamins (higher concentration was vitamin K 3.86 ppm) (Table 4). The peak area under the curve and the retention time of the standard and extracted phenols were used to determine the concentration and type of each component. The concentration for each compound was calculated as follows:

(4) $$\begin{array}{c} The\;concentration\;of\;the\;sample=\left(\frac{Area\;of\;the\;sample}{Area\;of\;the\;standard}\right)\times concentration\;of\;the\;standard\times \;\left(\frac{ml}{g}\right)\text{.} \end{array}$$

The results illustrated above were in agreement with many literature studies describing the analysis of the *T. aphylla* extract by HPLC. From these studies, they concluded that *T. aphylla* is a promising plant for many flavonoid compounds that have many pharmacological actions [25, 26].

#### 3.2.4. Effect of Sub-MIC Concentration for the Ethanol Extract on the Bacterial Adhesion Assay

**Figure 3:**
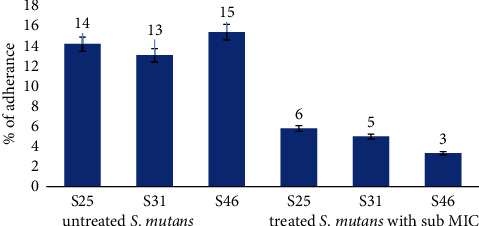
Comparison of the percentage of adherence of treated and untreated *S. mutans* with sub-MIC concentration. *P* value < 0.05.

Results performed in Figure 3 show a significant reduction in adherence to the glass surface after treating the samples with sub-MIC concentrations with the most affected isolate being S46 that reduced the adherence percentage from 15% to 3%.

This suggests that the *T. aphylla* extract may potentially be used to inhibit the proliferation of *S. mutans* and silence the expression of pathology-related genes, which will prevent the development of dental caries [27].

#### 3.2.5. Effect of Sub-MIC Concentration on *SpaP* Gene Expression

**Figure 4:**
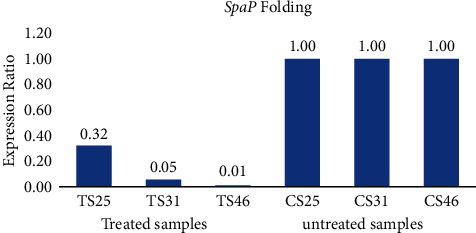
Comparison of the gene expression after and before treatment with sub-MIC concentration of the *T. aphylla* ethanolic extract.

To gain insight into adherence-related gene expression, real-time PCR analysis was used to quantify the effect of sub-MIC concentration of the *T. aphylla* ethanolic extract on the adherence of *S. mutans* (Table 5). Real-time PCR analysis was performed to evaluate the effect of different compounds on the gene expressions of virulence factors in *S. mutans*. Differences in the expression of virulence genes provided information on their function and helped in understanding the process. Our results showed that the expressions of the *SpaP* gene were downregulated in the presence of sub-MIC concentration of the *T. aphylla* ethanolic extract (Figure 4).

PCR is the best method of detection and diagnosis for various microorganisms [28]. This is in agreement with the finding of al-Ansari and his colleagues, which indicated that the gene regulation of treated *S. mutans SpaP* (survival promoter genes) when inhibited results in growth inhibition of *S. mutans* [29]. The surface protein antigen 1/11 gene (*SpaP*) is a housekeeping gene in *S. mutans* that interacts with the glycoprotein found in saliva to decide the receptors for the adhesion with teeth or gums, and its inhibition prevents *S. mutans* attachment with the teeth or gum of the host body [30]. The *S. mutans SpaP* gene is responsible for the survival of *S. mutans* in odd oral cavity ambiance [31].

The activity of 4-hydroxybenozic acid was improved by Kang and his colleagues in 2020 when they studied a significant virulence factor in the infection pathway of *Pseudomonas syringae* pv. tomato DC3000 (*Pst* DC3000), a type III secretion system (T3SS). To translocate effector proteins into host cells, which play a variety of roles in pathogenesis, the pathogen builds a type III apparatus. From *Sedum middendorfianum* root extract, 4-hydroxybenozic acid and vanillic acid were found to have an inhibitory effect on the promoter activity of the *hrpA* gene, which codes for the structural protein of the T3SS apparatus. Without affecting *Pst* DC3000's development, the phenolic acids at 2.5 mM dramatically reduced the expression of *hopP1*, *hrpA*, and *hrpL* in the *hrp/hrc* gene cluster. Treatment with 4-hydroxybenzoic acid and vanillic acid inhibited the autoagglutination of *Pst* DC3000 cells, which is triggered by T3SS [32].

## 4. Conclusions

From the abovementioned results, we can conclude that *Tamarix aphylla* extracts contain several components with significant medical importance. The *Tamarix aphylla* ethanolic extract exhibits a significant antimicrobial activity against multidrug-resistant*Streptococcus mutans*. The *Tamarix aphylla* ethanolic extract has a significant reduction in *SpaP* gene expression.

## Figures and Tables

**Table 1:** Primers used in this study.

Primer name	Sequence 5′-3′	Vol. of nuclease-free water (*μ*l)	Conc. (pmol/*μ*l)	Annealing temp. (°C)	Product size (bp)
SpaP-F	GACTTTGGTAATGGTTATGCATCAA	300	100	**60**	**101**
SpaP-R	TTTGTATCAGCCGGATCAAGTG	300	100		
16SrRNA	CCTACGGGAGGCAGCAGTAG	300	100		
16SrRNA	CAACAGAGCTTTACGATCCGAAA	300	100		

These numbers are taken from NCBI and another references.

**Table 2:** Retention time and the area of control and ethanol extracts of flavonoid.

Compound name	Reten. time (min)	Area (mV.s) control	Concentration control (ppm)	Area (mV.s) sample	Concentration sample (ppm)
4-Hydroxybenzoic	9.98	146.98	20	147.23	40.07
Cinnamaldehyde	10.40	500.40	10	673.46	26.92
Kaempferol	5.33	1248.96	10	837.12	13.40
Catechol	8.46	1031.10	20	303.93	11.79
Chlorogenic acid	17.14	922.28	20	197.20	8.55
Cinnamic acid	8.08	1405.17	10	385.95	5.49
Eugenol	13.82	4642.77	10	737.99	3.18
Quercetin	10.10	945.58	5	268.59	2.84
Pyrogallol	2.81	7093.76	5	1257.00	1.77
Rutin	4.24	16954.34	20	205.01	0.48
Lignan	16.85	11434.44	10	237.97	0.42
Gallic acid	3.57	6256.59	5	206.03	0.33

**Table 3:** Retention time and the area of control and ethanol extracts of alkaloid.

Compound name	Reten. time (min)	Area (mV.s) control	Concentration (ppm)	Area (mV.s) sample	Concentration (ppm)
Amfepramone	6.74	324.81	10	1099.93	67.73
Tropane	16.28	1722.10	10	1087.50	12.63
Thebaine	17.54	1796.21	10	1071.02	11.93
Berberine	14.75	805.85	10	473.94	11.76
Benzphetamine	7.34	790.08	10	401.48	10.16
Phencyclidine	8.46	865.10	10	293.66	6.79
Solanum	18.62	971.96	10	195.49	4.02
Caffeine	23.83	947.22	10	169.26	3.57
Gallocabechin	7.98	4729.78	10	650.19	2.75
Ethylbenzhydramine	10.52	6364.18	10	745.15	2.34
Veratrum	16.87	4624.97	10	539.65	2.33
Retrorsine	19.17	930.52	10	92.57	1.99
Pilocarpine	18.48	1209.29	10	111.20	1.84

**Table 4:** Retention time and the area of control and ethanol extracts of vitamin.

Compound name	Reten. time (min)	Area (mV.s) control	Concentration (ppm)	Area (mV.s) sample	Concentration (ppm)
K	13.24	3873.75	5	1494.10	3.86
E	6.87	1164.70	10	146.33	2.51
A	9.44	836.87	5	40.04	0.48
D3	7.59	26713.83	12.5	70.91	0.07

**Table 5:** *SpaP* gene expression after and before treatment with sub-MIC concentration of *T. aphylla* ethanolic extracts.

Samples	16SrRNA	*SpaP*	∆CT	∆∆CT	Folding
TS25	17.94	34.42	16.48	1.65	0.32
TS31	14.42	34.43	20.01	4.23	0.05
TS46	14.00	34.20	20.20	7.24	0.01
CS25	11.25	26.08	14.84	0.00	1.00
CS31	11.43	27.21	15.78	0.00	1.00
CS46	10.72	23.67	12.95	0.00	1.00

## Data Availability

All data are available and can be provided by the corresponding author upon request.
